# Supplementary figures and images for: Mitogenomic Codon Usage Patterns of Superfamily Certhioidea (Aves, Passeriformes): Insights into Asymmetrical Bias and Phylogenetic Implications
Source: Animals (Basel). 2022 Dec 27;13(1):96. doi: 10.3390/ani13010096 (PMC9817927; doi:10.3390/ani13010096)

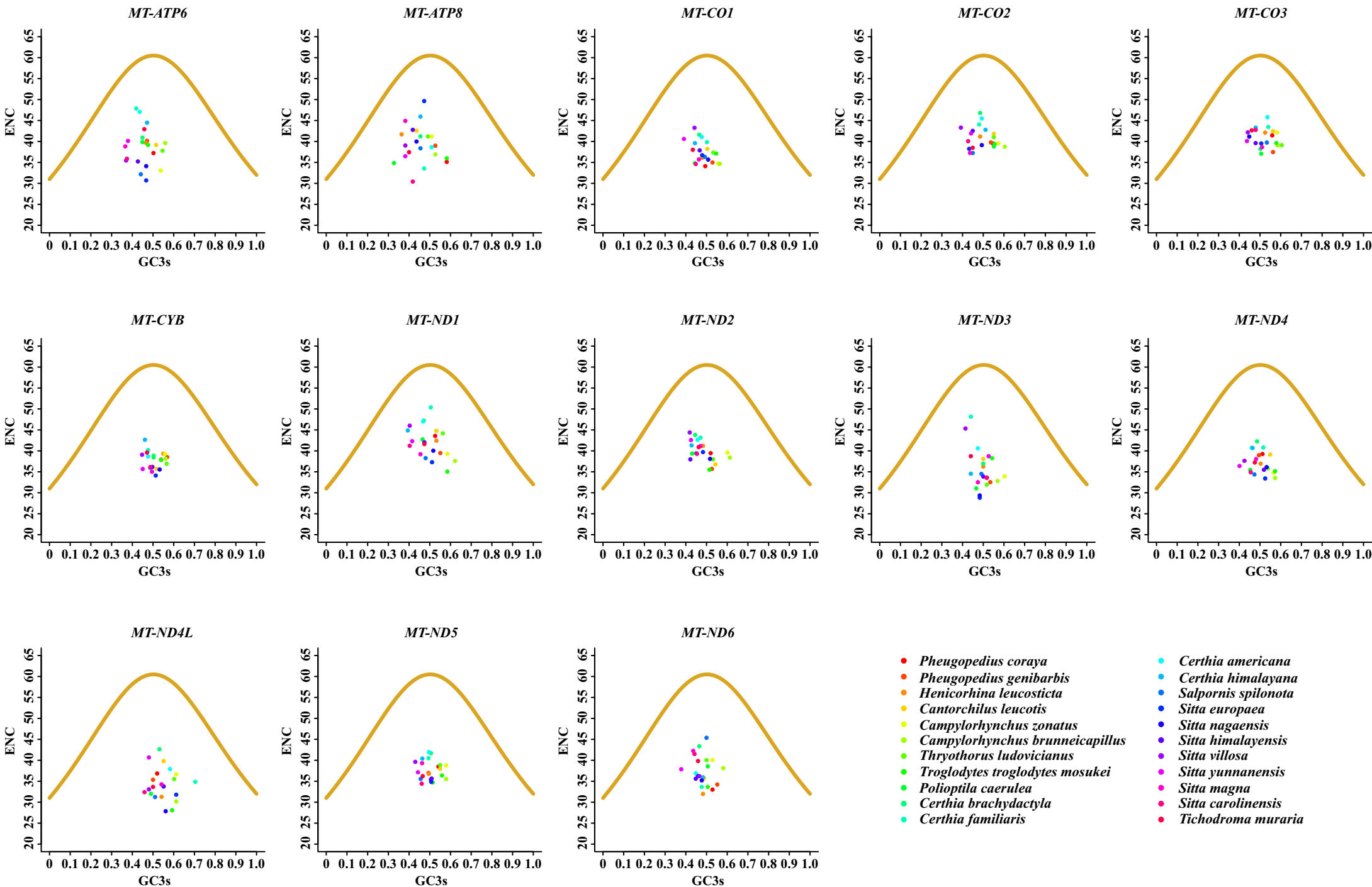

Supplement: Supplementary file 1 [file animals-13-00096-s001.zip › Figure S1.pdf]
